# 262 Voyages Beneath the Sea: a global assessment of macro- and megafaunal biodiversity and research effort at deep-sea hydrothermal vents

**DOI:** 10.7717/peerj.7397

**Published:** 2019-08-06

**Authors:** Andrew D. Thaler, Diva Amon

**Affiliations:** 1 Blackbeard Biologic: Science and Environmental Advisors, St. Michaels, MD, USA; 2 Center for Environmental Science, Horn Point Laboratory, University of Maryland, Cambridge, MD, USA; 3 Department of Life Sciences, Natural History Museum, London, UK

**Keywords:** Seafloor massive sulphide, Sampling effort, Benthos, Deep-sea mining, Chemosynthetic ecosystems, Western Pacific, East Pacific Rise, Mid-Atlantic Ridge, Indian Ocean, Southern Ocean

## Abstract

For over 40 years, hydrothermal vents and the communities that thrive on them have been a source of profound discovery for deep-sea ecologists. These ecosystems are found throughout the world on active plate margins as well as other geologically active features. In addition to their ecologic interest, hydrothermal vent fields are comprised of metallic ores, sparking a nascent industry that aims to mine these metal-rich deposits for their mineral wealth. Here, we provide the first systematic assessment of macrofaunal and megafaunal biodiversity at hydrothermal vents normalized against research effort. Cruise reports from scientific expeditions as well as other literature were used to characterize the extent of exploration, determine the relative biodiversity of different biogeographic provinces, identify knowledge gaps related to the distribution of research effort, and prioritize targets for additional sampling to establish biodiversity baselines ahead of potential commercial exploitation. The Northwest Pacific, Southwest Pacific, and Southern Ocean biogeographic provinces were identified as high biodiversity using rarefaction of family-level incidence data, whereas the North East Pacific Rise, Northern East Pacific, Mid-Atlantic Ridge, and Indian Ocean provinces had medium biodiversity, and the Mid-Cayman Spreading Center was identified as a province of relatively low biodiversity. A North/South divide in the extent of biological research and the targets of hydrothermal vent mining prospects was also identified. Finally, we provide an estimate of sampling completeness for each province to inform scientific and stewardship priorities.

## Introduction

When the *RV Knorr* set sail for the Galapagos Rift in 1977, the geologists aboard eagerly anticipated observing a deep-sea hydrothermal vent field for the first time. What they did not expect to find was life—abundant and unlike anything ever seen before. A series of dives aboard the *HOV Alvin* during that expedition revealed not only deep-sea hydrothermal vents but fields of clams and the towering, bright red tubeworms that would become icons of the deep sea. So unexpected was the discovery of these vibrant ecosystems that the ship carried no biological preservatives. The first specimens from the vent field that would soon be named “Garden of Eden” were fixed in vodka from the scientists’ private reserves (Ballard, 2000).

Since that first discovery, deep-sea hydrothermal vents have been found throughout the world’s oceans at mid-ocean ridges, volcanic arcs, and back-arc spreading centers (Beaulieu et al., 2013). As more geographic regions are explored, newly discovered vent fields present the potential for entirely new ecosystems as well as species. Vents can range from black smokers to shimmering diffuse flow, can exist on ultra-slow to ultra-fast spreading centers, can be talc-hosted, serpentinite-hosted, or any of several other geologic conditions (Van Dover, 2000). Just as “forest” can describe ecosystems ranging from boreal forests to tropical rain forest, “hydrothermal vent” describes a suite of deep-ocean ecosystems united by a shared dependence on chemosynthetically derived primary production and above-ambient temperatures but diverse in their composition and connection to one another.

While the hydrothermal vent fields discovered in the Galapagos Spreading Center and East Pacific Rise are dominated by the deep-sea tubeworm, *Riftia pachyptila*, subsequent vent ecosystems are characterized by swarms of shrimp (*Rimicaris exoculata* on the Mid-Atlantic Ridge: Dover et al., 1988; *Rimicaris hybisae* on the Mid-Cayman Spreading Center: Plouviez et al., 2015), aggregations of large snails (*Alviniconcha* spp. and *Ifremeria hessleri* in northern and southern West Pacific back-arc basins: Desbruyères, Hashimoto & Fabri, 2006, though *Alviniconcha* spp. are also found in the Indian Ocean: Suzuki et al., 2005), and colonies of yeti crabs (*Kiwa tyleri* in the Southern Ocean: Rogers et al., 2012). Many other biomass-dominant taxa have been recorded at vents, including a variety of tubeworms, mussels, clams, crabs, shrimp, snails, barnacles, and squat lobsters (Van Dover, 2000), but rare and often difficult to observe species also contribute to the biodiversity of a hydrothermal vent ecosystem (Mullineaux et al., 2018).

The discovery of large chemosynthetic communities around deep-sea hydrothermal vents fundamentally altered our understanding of how life endures in extreme environments (Rothschild & Mancinelli, 2001), the role of chemosynthesis in both exotic and mundane ecosystems (Boston, Ivanov & McKay, 1992; Dubilier, Bergin & Lott, 2008), and the role of symbioses in biological systems (Cavanaugh et al., 1981; Duperron et al., 2006). The discovery of other chemosynthetic cognate communities in the deep sea (e.g., methane seeps, mud volcanoes, whale falls, and wood falls: Van Dover, 2000) as well as terrestrial and shallow-water analogs (e.g., thermal springs: Yang et al., 2011 and the anoxic sediments of salt marshes: Howarth, 1984) followed.

While the scientific, ecological, societal, and cultural value of hydrothermal vent ecosystems is difficult to quantify (Thurber et al., 2014; Turner et al., 2019), the financial value of vent systems, and the ores they contain, is increasingly driving exploration in the deep sea. Over the last several decades, a new industry has emerged to explore the potential of mining Seafloor Massive Sulfides (or Polymetallic Sulfides)—deep-sea hydrothermal vents that contain high concentrations of rare and precious metals (Petersen et al., 2016). Though the deep-sea mining is in its infancy, multiple enterprises are developing mining prospects that include both active and inactive deep-sea hydrothermal vent fields.

Seafloor mineral resources in areas beyond national jurisdictions are managed by the International Seabed Authority (ISA) who adopted Regulations on Prospecting and Exploration for Polymetallic Sulfides in the Area in 2010. The ISA requires mining contractors to establish environmental baselines and maintain an environmental monitoring program before, during, and after operations (Bräger, Romero Rodriguez & Mulsow, 2018). Two international Codes of Conduct also apply to nations, organizations, and institutions who have voluntarily elected to abide by them. The InterRidge Statement of Commitment to Responsible Research Practices (Devey, Fisher & Scott, 2007) relates primarily to scientific research conducted at hydrothermal vents, including exploratory research to assess ore deposits. The International Marine Minerals Society Code for Environmental Management of Marine Mining establishes environmental principles and best practices for marine mining as well as recognizes the value of both biological and mineral resources (Verlaan, 2011). Additionally, the Intergovernmental Conference on Marine Biodiversity of Areas Beyond National Jurisdiction is working to establish legal instruments regulating the conservation and sustainable use of marine biological diversity in areas beyond national jurisdiction (Leary, 2019).

Within national waters (including territorial waters, exclusive economic zones, and the extended continental shelf), mining activities fall under national regulations, which vary depending on the country in question. Currently, Papua New Guinea, New Zealand, the Kingdom of Tonga, Japan, and Vanuatu have issued exploration permits to assess the value of ore found at deep-sea hydrothermal vents within their territorial waters (Boschen et al., 2013). In addition, Papua New Guinea has issued a single mining license for the resource-rich Solwara I hydrothermal vent field (Hoagland et al., 2010). As scientists, managers, and conservationists rush to establish best management practices ahead of proposed mining projects (Collins et al., 2013; Van Dover, 2010, 2011), a major challenge lies in our relatively limited knowledge of hydrothermal vent communities and our understanding of how these communities might respond to catastrophic anthropogenic disturbance.

Among the most pernicious problems in establishing a global assessment of biodiversity at deep-sea hydrothermal vents is understanding variation in the distribution of research effort across the oceans. Research has mostly focused on a few biogeographic provinces with multiple expeditions, long-term time-series, and cabled observatories, while other provinces have only recently been explored or sparingly sampled on few expeditions. In an ideal system, environmental managers would be able to draw from comprehensive, thorough, and rigorously tested sampling regimes to obtain clear, robust estimates of species richness and biodiversity. That is rarely the case, and is even less attainable in the deep sea, where accessing the seafloor is sporadic, opportunistic, and driven by inconsistent resource availability. Managers, regulators, and mining companies are working from incomplete data, with inferences about the consequences of human activity, as well as mitigation and remediation practices, often drawn from studies of few vent ecosystems that are often different from those in which the impacts are expected to occur. This is especially challenging as biodiversity is frequently used as a proxy for resilience and as a metric for assessing biological baselines (Sonter, Ali & Watson, 2018; Van Dover, 2010; Van Dover et al., 2017). In the absence of comprehensive standard sampling regimes, opportunistic data has to serve as a proxy when examining ecosystems threatened by commercial exploitation.

**Figure 1:**
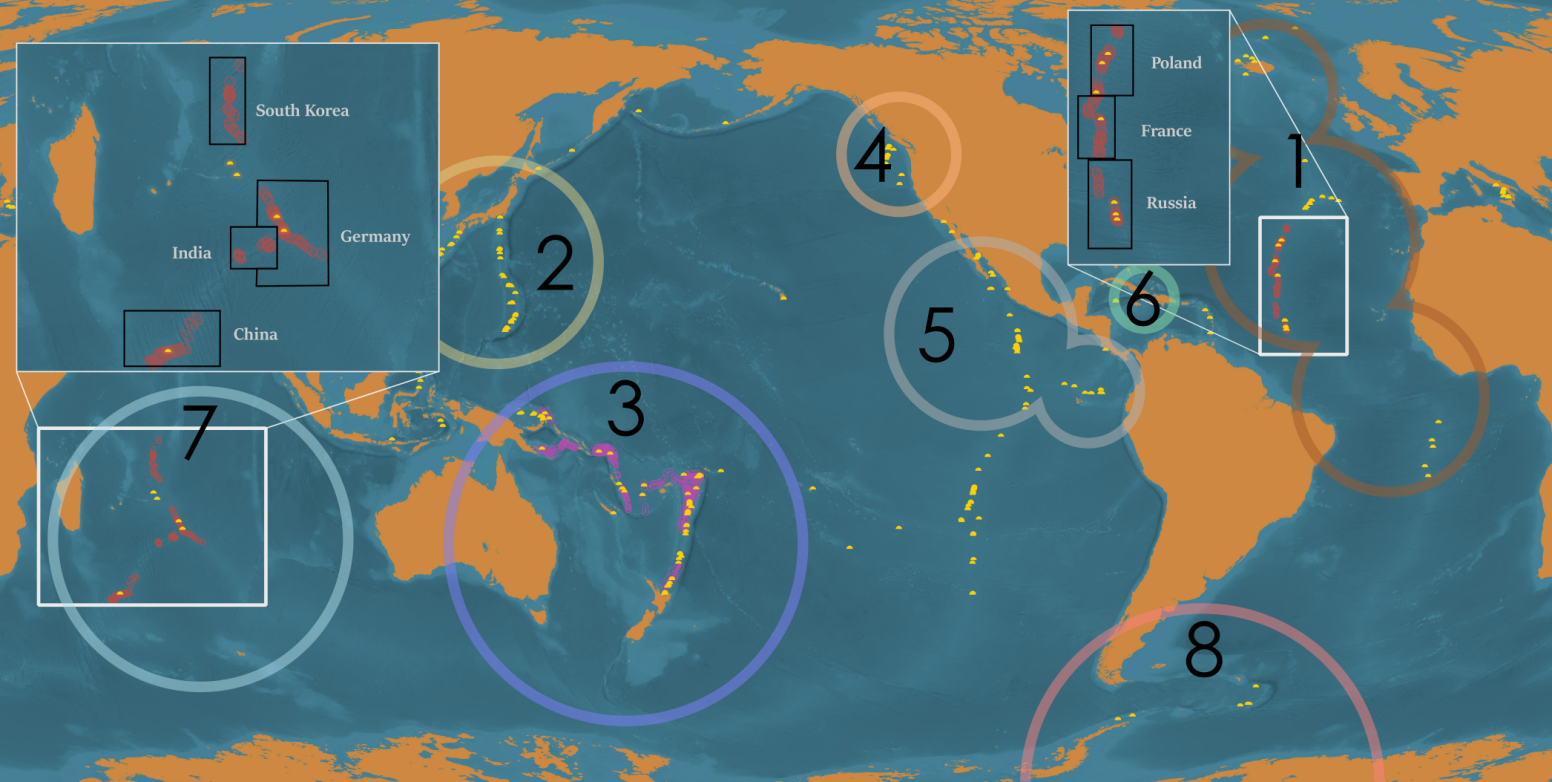
Global distribution of deep-sea hydrothermal vents, ISA-issued high seas mining exploration leases, and mining exploration licenses issued within territorial waters. Global distribution of active, confirmed deep-sea hydrothermal vents (yellow domes), ISA-issued mining exploration leases in areas beyond national jurisdiction (ABNJ) (red circles; note, bounding area is exaggerated for clarity), and mining exploration licenses issued within territorial waters (pink circles; note, bounding area is exaggerated for clarity). Black boxes indicate the member nations sponsoring claims in the area. White borders in inset represent exclusive economic zones. Large circles represent each biogeographic province for which sufficient data was available for analysis, in descending order of number of research cruises conducted in the region: (1) Mid-Atlantic Ridge, (2) Northwest Pacific, (3) Southwest Pacific, (4) Juan de Fuca Ridge, (5) Northern East Pacific Rise, (6) Mid-Cayman Spreading Center, (7) Indian Ocean, and (8) Southern Ocean. The Arctic, Mediterranean, and Southern East Pacific Rise biogeographic provinces are not indicated. Map prepared by Andrew Middleton.

In order to better assess our current understanding of deep-sea hydrothermal vent biodiversity, we undertook a survey of the last 40 years of vent research via cruise reports (post-research cruise summary documents that provide a day-to-day narrative of work at sea, as well as momentary sample logs and observations) from research expeditions that made biological observations at hydrothermal vents and compiled incidence data for macrofauna and megafauna sampled during these expeditions (Fig. 1). This allowed us to: assess and compare research effort among different biogeographic provinces; determine the relative biodiversity of biogeographic provinces when normalized against research effort; identify knowledge gaps related to the unequal distribution of research effort; and prioritize targets for additional sampling in advance of deep-sea mining.

## Methods

### Compiling cruise reports

To estimate global biological research effort at deep-sea hydrothermal vents, reports from discrete research expeditions were used as functional proxies for research effort. By assessing how many research cruises visited a particular region or vent system, we can gain a better understanding of the extent of global research effort and how that corresponds with both assessments of biodiversity as well as gaps in our knowledge for vulnerable hydrothermal vent ecosystems. For our purposes, “cruise reports” are considered any post-cruise literature that directly summarizes the activities conducted at sea. This can include narrative reports, observation logs from submersible or ROV surveys, or sample logs from shipboard sample processing. Importantly, cruise reports do not include the results of post-cruise analyses such as taxonomic studies or activities such as the publication of peer-reviewed manuscripts. This allows us to treat individual cruises as discrete sampling events reflective of the state of knowledge at or immediately after the time of sampling.

Cruise reports were acquired from institutional library archives, including those of Scripps Institute of Oceanography, Woods Hole Oceanographic Institution, Japan Agency for Marine-Earth Science and Technology (JAMSTEC), French Research Institute for Exploitation of the Sea, New Zealand National Institute of Water and Atmospheric Research (NIWA), Korea Institute of Ocean Science and Technology and others; regional databases, including rvdata.us and Natural Environment Research Council; identified through international collaborative databases, including InterRidge Cruise Database (https://www.interridge.org/IRcruise) and ChEssBase (Ramirez-Llodra & Blanco, 2005); and obtained through direct queries to chief scientists, principal investigators, and institutional archivists. In cases where cruise reports were not archived in English, we reached out to colleagues fluent in the appropriate languages to help identify records. In addition, we compiled data from each confirmed, active hydrothermal vent from InterRidge Vents Database Version 3.4 (https://vents-data.interridge.org).

Cruises were divided into 11 biogeographic provinces based on assessments by Bachraty, Legendre & Desbruyères (2009), Moalic et al. (2012), Rogers et al. (2012) and Suzuki et al. (2018). Those provinces consisted of the Arctic Ocean, Indian Ocean, Mediterranean Sea, Mid-Atlantic Ridge, Mid-Cayman Spreading Center, Northeast Pacific (which we refer to throughout as the Juan de Fuca Ridge to avoid confusion with the Northern East Pacific Rise, though other ridge axes are included in the assessment), Northern East Pacific Rise (including Galapagos Spreading Center), Southern East Pacific Rise (including Pacific-Antarctic Ridge), Southern Ocean, Northwest Pacific, and Southwest Pacific. Sampling effort was then assessed for each biogeographic province.

### Determining sampling effort

A comprehensive survey of all available cruise reports for biological sampling records from hydrothermal vent fields was undertaken. We define “biological sampling records” as either observation or collection and identification of macrofauna and megafauna while at sea. In cases where multiple ecosystems were observed or collected from during a single research cruise, we relied on narrative description and location records to determine whether those biological sampling records qualified as originating from a hydrothermal vent field. For ambiguous cases, we excluded those records.

Every research cruise has different objectives and sampling regimes, which influence how many and which taxa are sampled. To account for the high variability in sampling methodology, we recorded incidence, rather than abundance, data. We looked at macrofaunal and megafaunal occurrences, rather than microbial and meiofaunal (which are often preserved for post-cruise sorting and identification). We used identifications made at the time of sampling, rather than post-cruise analyses. In cases where the identification was unambiguous, but the taxonomic status of the organism has been revised since sampling (e.g., Siboglinidae, Pogonophora, and Vestimentifera: Pleijel, Dahlgren & Rouse, 2009) or clear and distinct common names were used prior to formal identification (e.g., the “Hoff Crab,” *K. tyleri*: Thatje et al., 2015 or “Scaly-foot Gastropod,” *Chrysomallon squamiferum*: Chen et al., 2015), the currently accepted nomenclature, as established by the World Register of Marine Species (Costello et al., 2013), was used. Organisms were documented to the lowest available taxonomic level and reported as present when identified in a cruise report.

### Biodiversity estimates

To estimate biodiversity across a global, inconsistent, and incomplete data set, incidence data was compiled at the taxonomic level of Family. Family richness has been shown to strongly correlate with species and genus richness in macroinvertebrates, especially in regions with relatively low species diversity (Heino & Soininen, 2007) and is useful in cases where sample sizes are inconsistent (Raup, 1975). Each cruise report was treated as a discrete sample for the purpose of this study.

The non-parametric asymptotic species richness estimator Chao2 (Chao et al., 2005) was used to extrapolate family richness of incidence data compiled from each research cruise. Chao2 has been demonstrated to be among the most reliable richness estimators when tested against simulated and real-world incomplete data sets where sampling effort is inconsistent (Walther & Morand, 1998). The bias-corrected Chao2 formula was used except in cases where the coefficient of variation for incidence distribution was greater than 0.5, in which case the classic formula was used following recommendations of Chao et al. (2005). Rarefaction estimates were extrapolated to twice the size reference sample, with 10,000 replicates randomized without replacement. Rarefaction extrapolations were also projected out to asymptote. EstimateS (version 9.1.0) was used to calculate Chao2, extrapolate rarefaction curves, and generate 95% confidence intervals (CIs) (Colwell, Coddington Jonathan & Hawksworth David, 1994; Colwell & Elsensohn, 2014).

Family richness estimates were referenced against the ChEssBase taxonomic archive, which contains self-reported identification of species from marine chemosynthetic ecosystems as an imperfect control (Ramirez-Llodra & Blanco, 2005). Though comprehensive at the time, ChEssBase is no longer being updated and does not contain observations recorded after April 2006. For biogeographic provinces in which sufficient cruise report data was available, the same rarefaction analyses were performed on a subset of cruise reports ending in 2005 to more accurately compare ChEssBase data with data derived these documents.

A secondary analysis was performed using methods outlined in Chao et al. (2009) and implemented in Microsoft Excel using a template provided in Chao et al. (2009) to estimate how many additional samples were needed for each biogeographic province to reach 80%, 90%, and 99% sampling completeness. This method can guide researchers and managers as to how much additional sampling is necessary in order to account for rare species within a biogeographic province and also inform management criteria in determining the proportion of completeness that satisfies the need for adequate biodiversity baseline assessments.

### Caveats and limitations

These samples are neither random nor even, but represent opportunistic sampling driven by a priori scientific priorities. While a study of this nature should not be used to derive fundamental ecologic principals, the use of opportunistic data within a management and conservation framework is essential for assessing the state of the field, determining priorities for future studies, and identifying knowledge gaps and data deficiencies. Rarefaction and extrapolation based on opportunistic samples has been used to guide conservation decision-making at local (Carvalheiro et al., 2013) and global scales (Maes et al., 2015).

While family richness has proven a useful proxy in some specific cases, higher level taxa are not always good proxies for species level diversity (Rosser, 2017) as higher taxa of the same rank (e.g., families) are not necessarily comparable across phyla (Gaston, 2000). To assess this potentiality, we independently analyzed family richness within phyla from a subset of research cruises to test whether there was variation in family accumulation between phyla.

Due to the variety of naming conventions, the incompleteness of the global research record, and inconsistencies within and among institutions, it is likely impossible to account for every research cruise that made biological observations at a deep-sea hydrothermal vent. Research archives may, in some cases, be incomplete and the quality of documentation and sample archiving at sea renders some cruise reports unsuitable for this study. In addition, geopolitical forces often shape international collaboration, and many documents are not necessarily available to the scientific community due to classified, proprietary, or privileged information.

The variable quality and completeness of cruise reports means that we expect this assessment to represent a minimum-viable approximation of biodiversity and sampling effort at deep-sea hydrothermal vents. Many deep-sea species are frequently undescribed at the time of sampling, and samples from more well-studied regions are more likely to be fully characterized to the species level, while samples from relatively understudied regions may be relegated to higher taxonomic identifications or characterized as undescribed at the time of sampling. This provides an additional challenge as rare species are far more frequently new-to-science and undescribed while common species are much more thoroughly identified.

To better assess the completeness of the sample set, we performed additional rarefaction extrapolation for a subsample of cruises conducted before 2006 to compare family richness estimated from the cruise reports to known family counts from the ChEssBase database. As samples are processed, identified, and archived to higher resolution post-cruise, we would expect the reported family counts from the database to be higher than those extrapolated directly from cruise reports. The ChEssBase database also includes all chemosynthetic ecosystems and neighboring ecosystems, as well as organisms sampled in the water column above a hydrothermal vent field, but not directly connected to the vents. This may artificially inflate family counts from ChEssBase.

Family incidence data and an archive of cruise reports are provided as Supplemental Data.

## Results

### Summary of cruise reports

**Figure 2:**
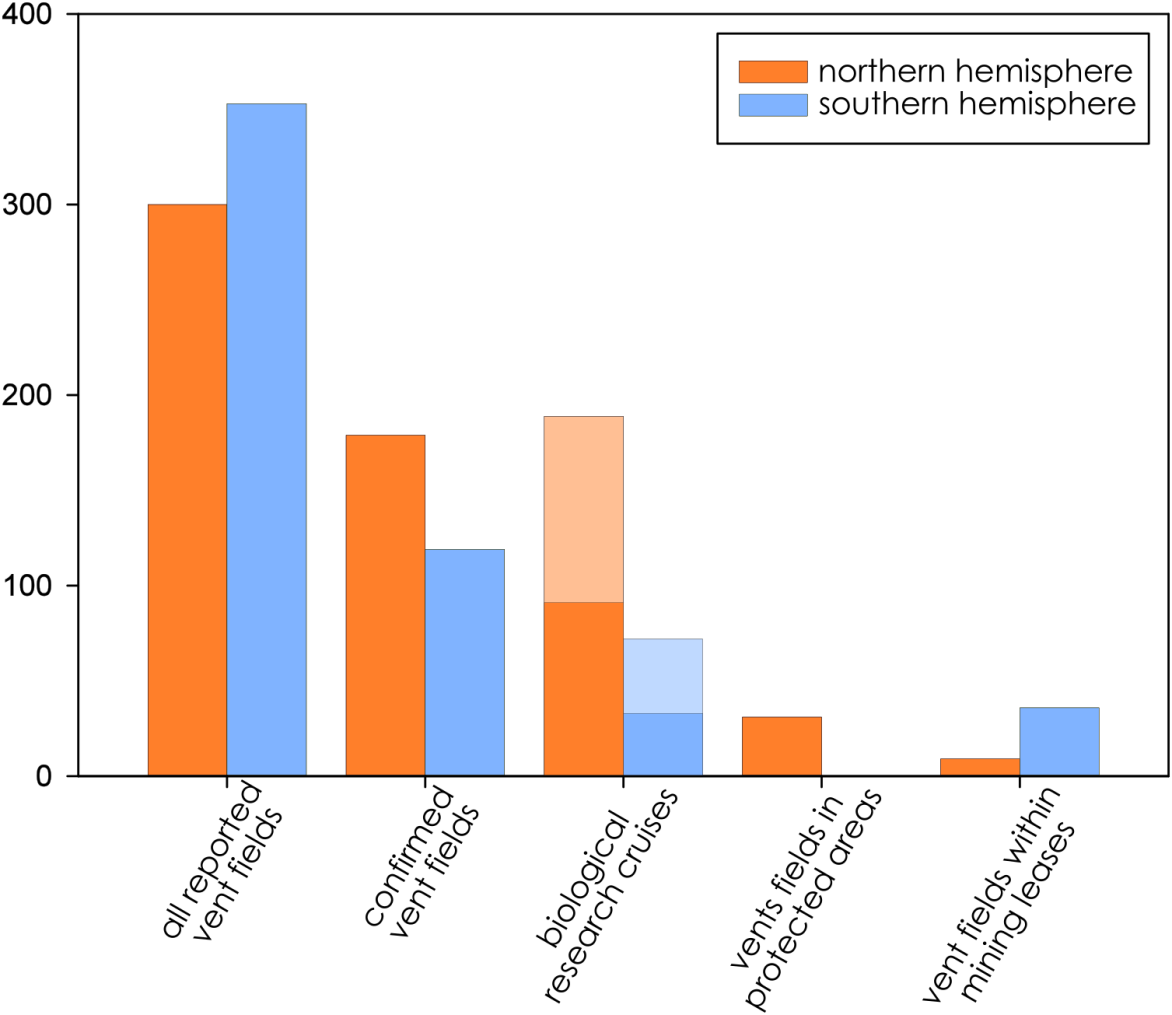
Distribution of active hydrothermal vent field, biological research cruises (subset used for biodiversity analysis in dark colors overlaid on all documented research cruises), vent fields in protected areas, and vent fields that fall within mining leases.

We identified a total of 262 research cruises representing 12 nations and one territory (Australia, Canada, France, Germany, Ireland, Japan, New Caledonia, New Zealand, Portugal, Russia or the Soviet Union, South Korea, the UK, and the USA) that collected biological samples or made biological observations at deep-sea hydrothermal vents spanning from 1977 to 2017 (Fig. 2). Of those, 124 had cruise reports of sufficient detail for biodiversity analysis.

Of the 841 research cruises identified through the InterRidge archive (https://www.interridge.org/IRcruise), 88 contained sufficiently detailed biological sampling based on available cruise reports, while the remainder were geologic, geophysical, oceanographic, or exploration-related without a documented biological component or without detailed sample logs and dive narratives. A total of 179 research cruises were identified through archive requests, references in other literature, contributions from principle investigators and cruise participants, and other documentation (Table 1). Notably absent were many Soviet-era cruises from the former USSR, the reports of which could not be located by colleagues, as well as cruises conducted by both national and corporate interests for the purposes of mining exploration, which are generally held as proprietary information. Research cruises from the late 1970s and early 1980s were less well documented and full cruise reports could not be located for several early research expeditions, particularly to the Galapagos Spreading Center.

**Table 1:** Summary of distribution of hydrothermal vent field, research effort, and families identified from cruise reports and databases by biogeographic province.

Biogeographic Province	Active, confirmed vent fields	Vent fields in EEZs	Vent fields in ABNJ	Vent fields in mining leases	Research cruises	Available cruise reports	Families sampled	ChEssBase families
Arctic	7	8	0	0	2	0	–	1
Indian Ocean	6	3	3	3	16	7	33	6
Mediterranean	15	15	0	0	3	0	–	0
Mid-Atlantic Ridge	27	14	13	9	61	27	53	119
Mid-Cayman Spreading Center	2	2	0	0	8	5	16	0
Northeast Pacific (Juan de Fuca Ridge)	20	9	11	0	34	14	16	86
Northern East-Pacific Rise	37	14	23	0	31	8	36	104
Southern East-Pacific Rise	29	27	2	0	4	3	61	69
Southern Ocean	6	2	4	0	5	5	23	1
Northwest Pacific	51	51	0	0	54	40	106	55
Southwest Pacific	68	67	1	33	44	16	54	56

**Note:**

Confirmed Vent Fields, Vent Fields in EEZs, Vent Fields in High Seas, and Vent fields in Mining Leases from Beaulieu et al. (2013). ChEssBase accessed via Ocean Biogeographic Database System (Ramirez-Llodra & Blanco, 2005).

Nearly a third of all available cruise reports were from research conducted in the Northwest Pacific (*n* = 40) due in no small part to the rigorous archiving conducted at JAMSTEC. The Mid-Atlantic Ridge (*n* = 27), Southwest Pacific (*n* = 16), and Juan de Fuca Ridge (*n* = 14) can also be considered extensively sampled systems. Surprisingly few cruise reports were available from the Northern East Pacific Rise (*n* = 8) despite a history of extensive sampling (we identified 31 biological research cruises to the Northern EPR). The Southern Ocean, Mid-Cayman Spreading Center, Indian Ocean, and Southern East Pacific Rise had the fewest available cruise reports (*n* = 7, 5, 5, 3 respectively). No cruise reports containing biological sampling data of macrofauna were available from the Mediterranean Sea or Arctic Ocean (Table 1). These two provinces, as well as the Southern East Pacific Rise, were excluded from subsequent analyses.

Biological research at deep-sea hydrothermal vents has been historically concentrated in the northern hemisphere (Fig. 2), while the majority of southern-hemisphere vents were only discovered in last 15 years with much fewer expeditions since (https://vents-data.interridge.org). Across all known hydrothermal vent fields (those that have been either directly confirmed or inferred based on chemical or geologic signals), a majority (353) occur in the southern hemisphere compared with 300 from the northern hemisphere. While of the 298 visually confirmed, active hydrothermal vent sites, 179 occur in the northern hemisphere and 119 are found in the southern hemisphere. More than twice times as many biologic research cruises have been undertaken in the northern hemisphere (189) than in the southern hemisphere (72). Among active vent fields that fall within exploratory or exploitation mining leases, a majority were located in the Southern Hemisphere (nine in the Northern Hemisphere; 36 in the Southern Hemisphere).

### Estimates of family richness

**Figure 3:**
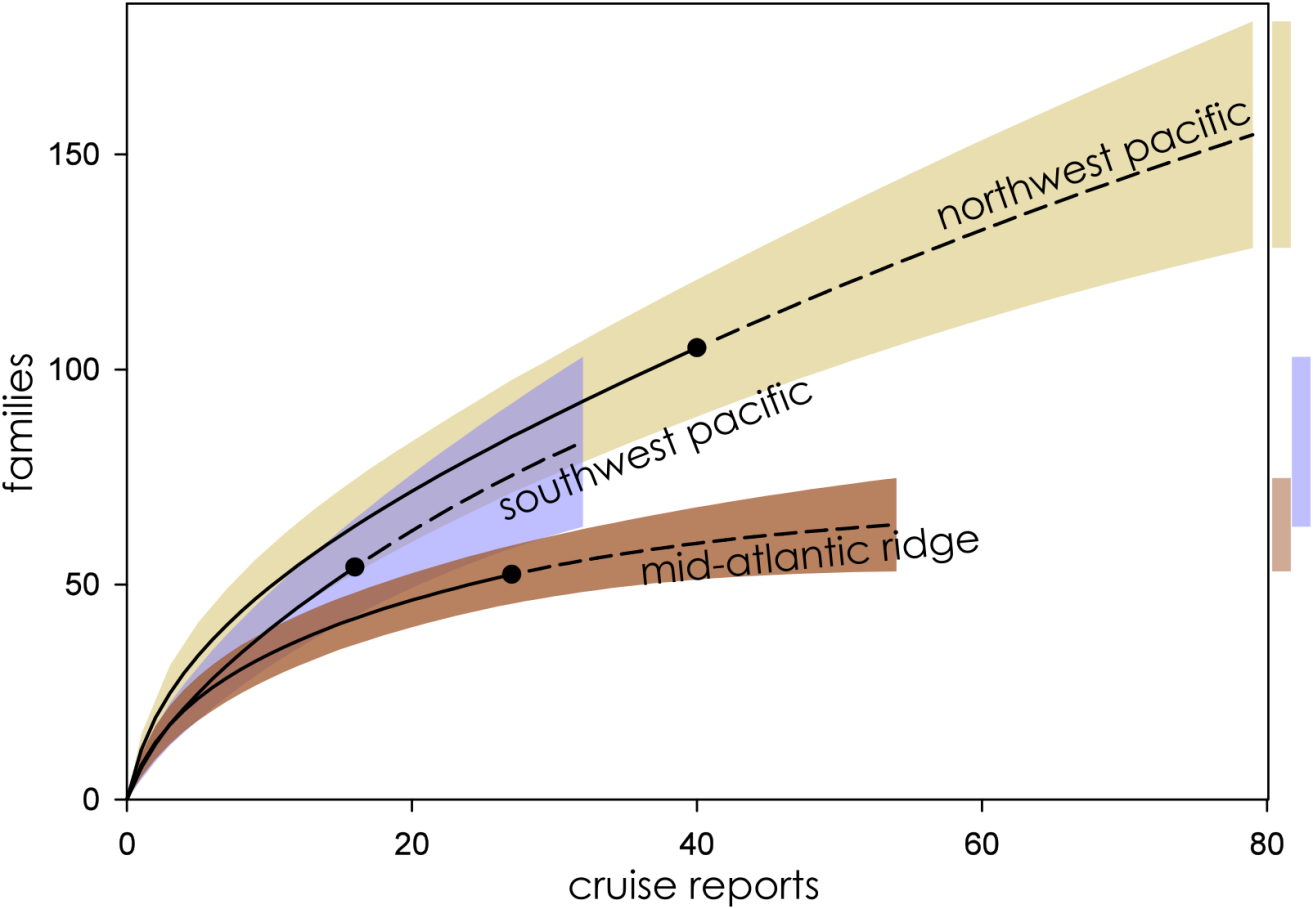
Family richness in the Northwest Pacific, Southwest Pacific, and Mid-Atlantic Ridge. Parametric interpolation (solid line terminating in black dot) and non-parametric asymptotic extrapolation (dashed line) with 95% confidence intervals (colored bounding areas). Color-coded guide bars on far right correspond to 95% confident intervals at twice the reference sample.

**Figure 4:**
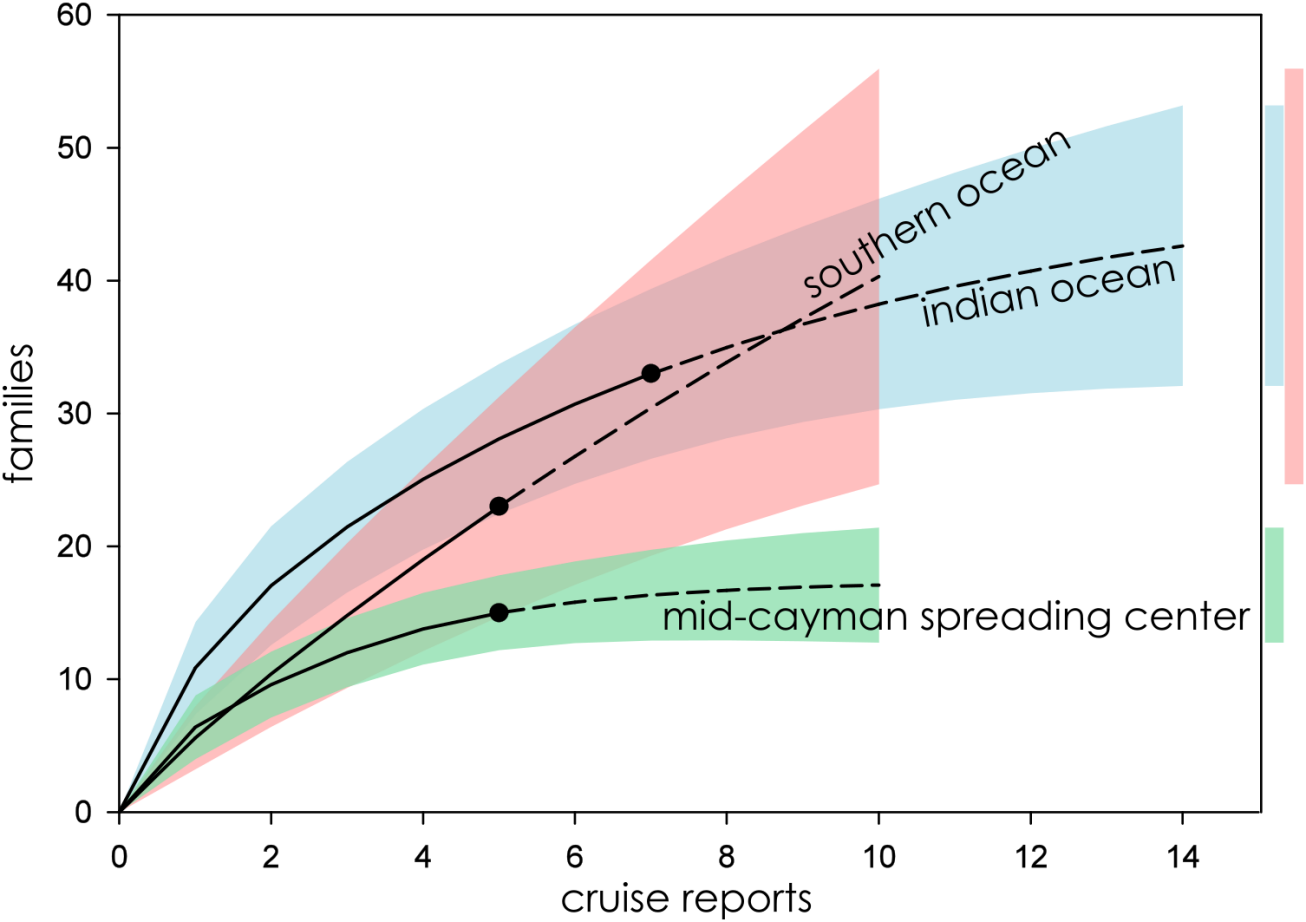
Family richness in the Southern Ocean, Indian Ocean, and Mid-Cayman Spreading Center. Parametric interpolation (solid line terminating in black dot) and non-parametric asymptotic extrapolation (dashed line) with 95% confidence intervals (colored bounding areas). Color-coded guide bars on far right correspond to 95% confident intervals at twice the reference sample.

**Figure 5:**
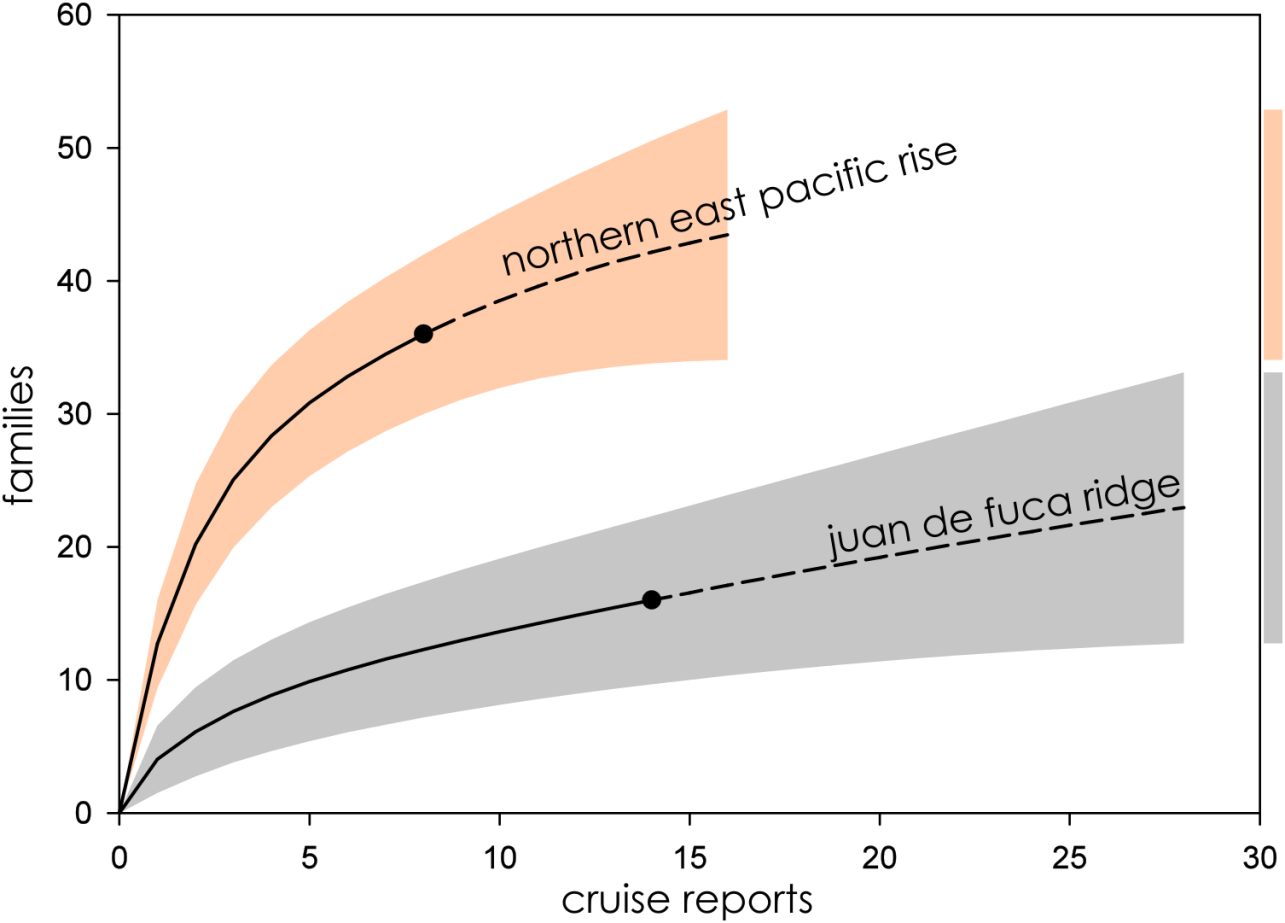
Family richness on the Northern East Pacific Rise and Juan de Fuca Ridge. Parametric interpolation (solid line terminating in black dot) and non-parametric asymptotic extrapolation (dashed line) with 95% confidence intervals (colored bounding areas). Color-coded guide bars on far right correspond to 95% confident intervals at twice the reference sample.

Eight biogeographic provinces had sufficient data available for analysis and were extrapolated out to twice the reference sample. Estimated family richness ranged from a high of 155.6 (Northwest Pacific; 95% CI [129.0.9–182.3]) to a low of 17.1 (Mid-Cayman Spreading Center; 95% CI [12.8–21.4]). The Northwest Pacific and Southwest Pacific (83.2; 95% CI [63.4–102.6]; Fig. 3), while the Southern Ocean (40.3; 95% CI [24.7–55.9]) though it contained fewer samples, shared a similar trajectory (Fig. 4). The Northern East Pacific Rise (43.48; 95% CI [34.1–52.9]) had greater family richness than the Juan de Fuca Ridge (23.0; 95% CI [12.8–33.1]) despite fewer available cruise reports (Fig. 5). The Mid-Atlantic Ridge (63.9; 95% CI [53.0–74.7]) and Indian Ocean (42.6; 95% CI [32.1–53.2] shared a similar pattern with the Northern East Pacific Rise, while the Mid-Cayman Spreading Center fell far below all other biogeographic provinces in family richness (Fig. 5).

**Figure 6:**
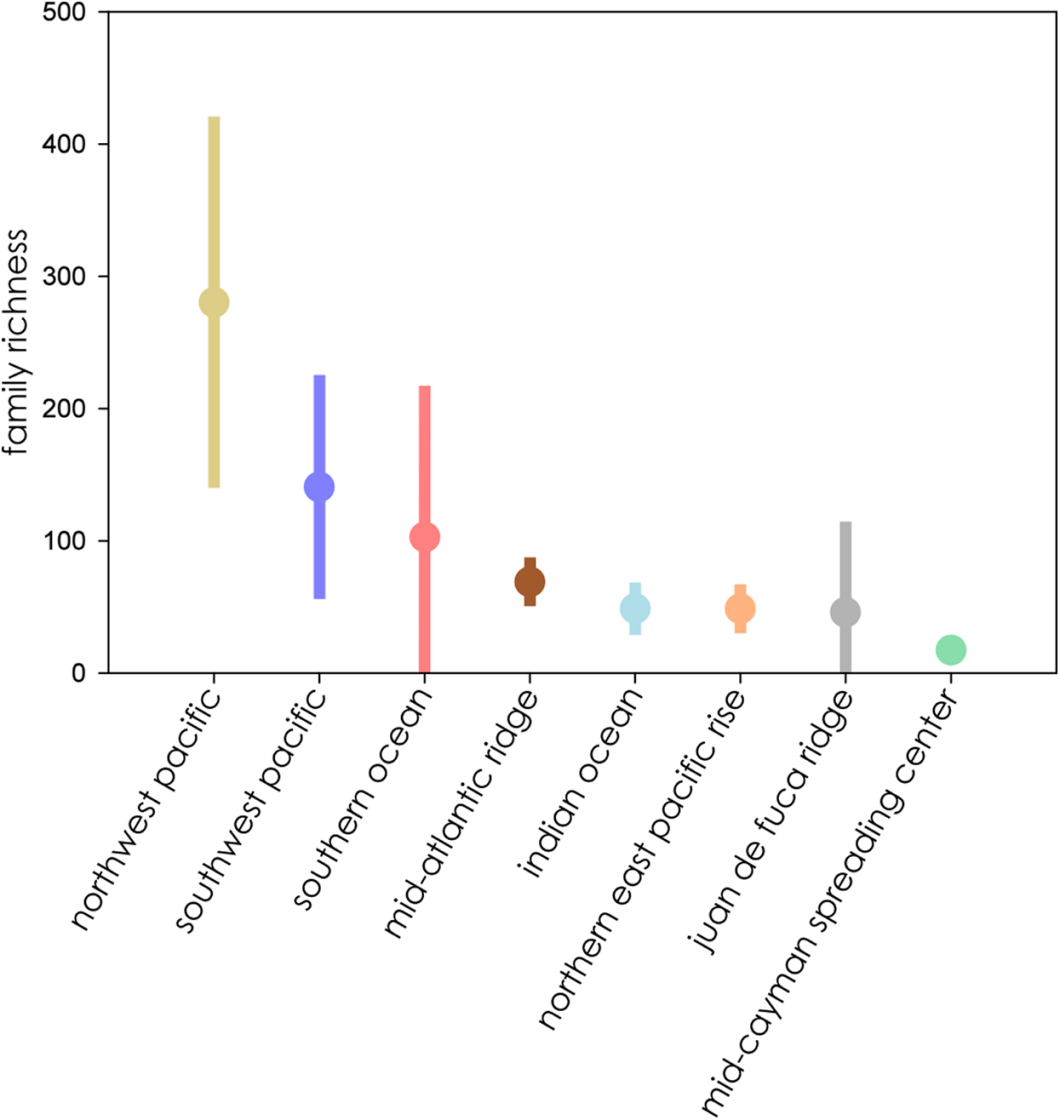
Family richness of all eight biogeographic provinces ranked by mean richness from highest (left) to lowest) where all extrapolations reached asymptote. Bars represent extent of 95% confidence intervals.

When ranked from highest to lowest biodiversity using extrapolation to asymptote for all biogeographic provinces, the Northwest Pacific had the highest mean family richness, followed by the Southwest Pacific, Southern Ocean, Mid-Atlantic Ridge, Indian Ocean, Northern East Pacific Rise, and Juan de Fuca Ridge, with the Mid-Cayman Spreading Center coming in a distant last (Fig. 6). The wide CIs for the Southern Ocean and Juan de Fuca Ridge that intersect with null make any placement tenuous given the current data.

The Mid-Atlantic Ridge, Northern East-Pacific Rise, and Southwest Pacific biogeographic provinces were further subdivided by phyla to better clarify the role that taxonomic variability plays in assessments of family richness. Annelids, arthropods, and mollusks were independently assessed for family richness. In all three cases, and for all three phyla, 95% CIs overlapped with no clear pattern of variation among family accumulation extrapolations (Fig. S1). For the Southwest Pacific, in particular, rarefaction curves for family accumulation were nearly identical. On the Mid-Atlantic Ridge, extrapolated annelid family accumulation approached an asymptote while arthropods and mollusks continued to rise with 100% overlap between CIs. On the Northern East-Pacific Rise, extrapolated arthropod family accumulation approached an asymptote while annelids and mollusks continued to rise with 100% overlap between CIs.

Comparisons between ChEssBase and raw cruise data revealed consistently low estimates. The Mid-Atlantic Ridge, Northern East-Pacific Rise, and Southwest Pacific all had sufficient cruise reports available to compare pre-2006 cruises to the database. Estimated number of families when extrapolated out to two times the sample size represented 40% (Mid-Atlantic Ridge), 36% (North East-Pacific Rise), and 67% (Southwest Pacific) of the total families reported in ChEssBase (Fig. S2). When extrapolated using the full dataset, family richness for the Mid-Atlantic Ridge accounted for 58% of the mean observed families in ChEssBase (73% of the maximum CI), family richness for the North East Pacific Rise accounted for 47% of the mean observed families in ChEssBase (64% of the maximum CI), and family richness for the Southwest Pacific accounted for 250% of the mean observed families in ChEssBase (401% of the maximum CI).

### Estimates of sample completeness

Using Chao’s method for estimating sample completeness, we were able to generate rough estimates of how many additional research cruises would be required to comprehensively sample each biogeographic province. A few provinces required less than 10 additional biological research cruises of comparable survey design to those examined in order to reach 90% sample completeness, including the Indian Ocean (9) and Northern East Pacific Rise (9). Perhaps not surprisingly, the Mid-Cayman Spreading Center was the closest to being comprehensively sampled (86% complete with 11 additional cruises required to reach unity). Other provinces required 10–100 additional research cruises, including the Mid-Atlantic Ridge (20), Juan de Fuca Ridge (99), Southern Ocean (43), and Southwest Pacific (71) to reach 90% sampling completeness. Despite being the most extensively sampled of all the biogeographic provinces, the Northwest Pacific required an additional 216 research cruises to reach 90% completeness. Of provinces that could be assessed, the three least sampled provinces with respect to their extrapolated biodiversity and estimates of sample completeness were the Southern Ocean (22% complete), Northwest Pacific (37% complete), and Southwest Pacific (38% complete). Three biogeographic provinces lacked sufficient samples for analysis.

## Discussion

Forty years after the discovery of deep-sea hydrothermal vents, these remote and inaccessible ecosystems continue to produce new insights and new discoveries. In the last decade, the number of known active vent fields has doubled and yet current estimates project that two thirds of all hydrothermal vent fields are still waiting to be discovered (Beaulieu, Baker & German, 2012). Since their discovery, two new species have been described, on average, each month from hydrothermal vents (Ramirez-Llodra, Shank & German, 2007). This rate of description is tempered by the fact that research effort has been, until recently, fairly narrowly focused on key biogeographic provinces in the northern hemisphere. Only a small fraction of all active ridge systems have been explored for hydrothermal activity (Baker & German, 2004; Beaulieu, Baker & German, 2015).

Biodiversity estimates clustered into three general overlapping groups. The biogeographic provinces with the highest estimated biodiversity were also among the most geologically diverse. The North- and Southwest Pacific and the Southern Ocean contain both arc and back-arc settings, while those with medium biodiversity, the Mid-Atlantic Ridge, Northern East Pacific Rise, Juan de Fuca Ridge, and Indian Ocean, occur on mid-ocean ridges. The lowest biodiversity province, the Mid-Cayman Spreading Center, occurs along a transform fault on a relic spreading center, far removed from other vent systems. Multiple studies have highlighted that vent distribution on back-arc basins is geographically complex compared to the more linear mid-ocean ridges, leading to patchy connectivity among vent fields, which can promote great biodiversity (Audzijonyte & Vrijenhoek, 2010; Vrijenhoek, 2010).

The Global South is frequently underrepresented in both terrestrial and marine ecological studies (Ladle et al., 2015; Martin, Blossey & Ellis, 2012; Velasco et al., 2015). This pattern is reinforced by several factors including the modern concentration of financial and educational resources in the northern hemisphere, a history of colonization and post-colonial exploitation, and a lack of representation within the scientific community (Doi & Takahara, 2016; Wilson et al., 2016) leading to a stark divide in the availability of comprehensive baseline surveys to make conservation and management decisions in at-risk ecosystems (Karlsson, Srebotnjak & Gonzales, 2007). It is clear that deep-sea research is not immune to this phenomenon.

### Northwest and Southwest Pacific

The highest extrapolated biodiversity among all biogeographic provinces was estimated in the Northwest Pacific, which also has the longest rising arc before reaching asymptote and the highest number of observed Families in the cruise reports (Fig. 2). Due to its proximity to JAMSTEC, the Northwest Pacific was among the best studied biogeographic provinces based on available cruise reports, and yet it had among the lowest *g*-values (Table 2) for estimated sampling completeness, suggesting a vast, unsampled reservoir of family-level biodiversity still waiting to be discovered.

**Table 2:** Chao estimates of sample completeness for hydrothermal vent ecosystems in eight biogeographic provinces.

Biogeographic Province	*T*	*Q*_1_	*Q*_2_	*t*	*F*_obs_	*g*	1.0	0.99	0.9	0.8
Indian Ocean	76	16	7	7	33	0.68	43	26	9	4
Mid-Atlantic Ridge	223	20	12	28	53	0.77	127	73	20	4
Mid-Cayman Spreading Center	32	6	6	5	15	0.86	11	7	1	–
Northeast Pacific (Juan de Fuca Ridge)	56	8	1	14	16	0.35	315	220	99	62
Northern East-Pacific Rise	102	12	5	8	36	0.74	49	29	9	3
Southern Ocean	28	20	2	5	23	0.22	150	90	43	28
Northwest Pacific	461	60	10	40	105	0.37	943	487	216	134
Southwest Pacific	117	36	7	16	54	0.38	286	162	71	44

**Note:**

*T* is the total number of presence records, *Q*_1_ is the number of observed singletons, *Q*_2_ is the number of observed doubletons, *t* is the total number of samples, *F*_obs_ is the observed number of families, *g* is the proportion of completeness. 1.0, 0.99, 0.9, and 0.8 are the relative completeness proportions from which the number of additional samples needed was inferred (as these values represent potential research cruises, they are rounded up to the nearest whole number).

The Southwest Pacific followed a similar trend, with half the observed families and a lower mean family richness. Sampling effort was similar to that of the Northwest Pacific. Far fewer cruise reports are available from the Southwest Pacific, with sampling predominantly focused around the Kermadec Arc, where NIWA is situated. A number of additional research cruises are known to have been conducted in this province, in particular around Manus Basin in Papua New Guinea, as well as other Pacific Islands, by mining companies (AD Thaler, 2019, personal observation). Those cruise reports and sample logs are proprietary and not publicly available.

Of the seafloor massive sulfide mining prospects currently in development, the two closest to commercial production lie in the Northwestern Pacific off the coast of Japan (Okamoto et al., 2018) and in the Southwest Pacific in the territorial waters of Papua New Guinea (Coffey Natural Systems, 2008). Collectively, the West Pacific represents a region of exceptional hydrothermal-vent biodiversity with tremendous potential for new discovery while simultaneously facing the most imminent threat from deep-sea mining of seafloor massive sulfides.

### Mid-Atlantic Ridge and Indian Ocean

Despite dramatically different sampling regimes, the Mid-Atlantic Ridge and Indian Ocean biogeographic provinces shared many characteristics in terms of distribution and extrapolated family richness. With relatively direct access from both western Europe and the United States’ east coast, and sustained attention from American and European research institutions, the hydrothermal vents on the Mid-Atlantic Ridge are among the most intensively studied of all the biogeographic provinces.

Meanwhile, hydrothermal vents in the Indian Ocean, likewise situated on a mid-ocean ridge, exhibited a lower mean family richness within overlapping CIs of roughly the same extent as those of the Mid-Atlantic Ridge. Though there has historically been less research focused on the Indian Ocean, this pattern, as well as a proliferation of novel species and taxa (e.g., *C. squamiferum*; Chen et al., 2015) and the growth of deep-sea research institutions in India and China suggests that hydrothermal vents in the Indian Ocean could play as significant a role in the exploration of the deep sea in this century as the Mid-Atlantic Ridge played in the last.

### Northern East Pacific Rise and Juan de Fuca Ridge

The Northern East Pacific Rise and Juan de Fuca Ridge presented a challenging case to assess, as there was a dearth of available cruise reports from these extensively studied regions. The relatively low completeness of the Juan de Fuca Ridge in particular is likely an artifact of these missing cruise reports, as ChEssBase lists many more taxonomic records than those uncovered through sampling effort analysis (Table 1). Due to the way data is compiled and queried in ChEssBase, this may represent an over-estimate, as ChEssBase includes all chemosynthetic ecosystems, including methane seeps which are also found in close proximity to hydrothermal vents in this region (Ramirez-Llodra & Blanco, 2005).

The Northern East Pacific Rise biogeographic province followed the same pattern of family accumulation and estimated family richness as other mid-ocean ridge systems, however, though estimated family richness is comparable to the Juan de Fuca Ridge, the wide CIs of the Juan de Fuca Ridge are more in line with back-arc basin vent ecosystems (Fig. 6). This furthers supports the interpretation that the region is undersampled, either practically or as a result of the low availability of reports from known research cruises.

### Southern Ocean and Mid-Cayman Spreading Center

Hydrothermal vent fields in the Mid-Cayman Spreading Center (Fig. 4) and in the Southern Ocean (Fig. 2) along the East Scotia Ridge provided a useful illustration of the variability within deep-sea vent communities. Both systems were only recently characterized—hydrothermal vents in the Mid-Cayman Spreading Center were first sampled in 2010 (Plouviez et al., 2015), while those of the East Scotia Ridge were first observed in 2009 (Rogers et al., 2012). Both vent fields represent new, albeit small, biogeographic provinces. And, conveniently, both vent systems were largely studied by the same personnel from the National Oceanography Centre, Southampton, using similar sample designs deployed using the same equipment, with many of the same taxonomists identifying taxa at sea (AD Thaler, 2019, personal observation). Five separate research cruises made biological observations and provided comprehensive sample logs for two discrete hydrothermal vent fields within each biogeographic province.

Despite nearly identical sampling effort, the Mid-Cayman Spreading Center exhibits the lowest macro- and megafaunal biodiversity of any known hydrothermal vent system, an observation that has been anecdotally expressed by numerous hydrothermal vent ecologists (A Glover, 2013, personal communication; J Copley, 2013, personal communication), while the Southern Ocean has among the highest family richness (though the 95% CI is quite wide). Completeness estimates (Table 2) indicate that, while the Mid-Cayman Spreading Center is approaching unity and is currently estimated to be among the best sampled hydrothermal vent systems in terms of estimated family richness, the Southern Ocean is the poorest sampled biogeographic province.

This comparison is particularly valuable, as it demonstrates that family richness estimates at deep-sea hydrothermal vents are not just an artifact of sampling effort but reflect real and observable differences in biodiversity among hydrothermal-vent biogeographic provinces.

### Arctic Ocean, Mediterranean Sea, and Southern East Pacific Rise

Three biogeographic provinces, the Arctic Ocean, Mediterranean Sea, and Southern East Pacific Rise had too little data available to appropriately assess sampling effort. While there are several know research cruises to the Arctic Ocean, in particular to the Loki’s Castle vent field along Mohn’s Ridge (Edmonds et al., 2003), the majority were conducted in conjunction with commercial resource exploration and their subsequent reports are not publicly available. As Loki’s Castle shares vent fauna from both the Atlantic and Pacific and appears to be dominated by an undescribed species of amphipod, as well as methane seep-associated tubeworms, it has the potential to represent an intermediate province that connects Atlantic and Pacific vent systems (Schander et al., 2010).

The Mediterranean Sea presents a very different story. Several recent expeditions have sampled microbes from hydrothermal vent fields in the Mediterranean Sea as well as siboglinid tubeworms that are closely related to those found on deep-sea methane seeps (Taviani, 2014), yet no vent-endemic fauna were observed. It is likely that, due to their relatively shallow depth (200–500 m), Mediterranean hydrothermal vent fields have not developed their own characteristic chemoautotrophic macrofaunal communities (Biasi & Aliani, 2003; Dando, Stüben & Varnavas, 1999; Danovaro et al., 2010).

Meanwhile, there are substantial biodiversity records from the Southern East Pacific available on ChEssBase, but few accessible cruise reports from the region. The current state of knowledge suggests that the Southern East Pacific allies closely with Northern East Pacific Rise, with significant dispersal barriers for some, but not all, co-occurring taxa, and its assignment as a separate biogeographic province may be premature (Jollivet et al., 2004; Plouviez et al., 2009, 2010; Rybakova & Galkin, 2015; Won et al., 2003).

### Limitations of available data

While this study provides a rough initial estimate of global hydrothermal vent biodiversity, it is necessarily incomplete. Despite over 250 documented research cruises undertaken to investigate the biology, ecology, and evolution of macro- and megafauna at deep-sea hydrothermal vents, we have barely begun to probe the surface of one of the world’s most remote and inaccessible ecosystems.

While Family-level richness has a mixed track-record as a proxy for species richness when estimating biodiversity, extrapolation of family richness at three biogeographic provinces when subdivided by phyla suggests that, in the case of hydrothermal vent ecosystems, family richness may serve as an appropriate proxy. Higher-level taxa work better as a proxy for species richness when the species to family ratio is relatively low (Rosser, 2017). Deep-sea hydrothermal vents are notable for having low biodiversity compared to other deep-sea ecosystems (Van Dover, 2000).

Comparisons with ChEssBase reveal a key limitation of using cruise reports to assess biodiversity: some organisms are easier to identify at sea while others require more involved identification on land. With the exception of the Southwest Pacific, observed families reported in ChEssBase exceeded estimated family richness, despite the database being over 10 years out of date. This is due in large part to continued efforts on land, once a cruise is complete, to identify and categorize sampled organisms that were not immediately cataloged onboard. While it would be ideal to include any post-cruise identification, the practical and collaborative nature of deep-sea taxonomy means that new species categorized post-cruise can be, and often are, identified through multiple samples derived from many cruises. These confound the assumption of a research cruise as a discrete sampling unit and their inclusion is beyond the scope of this study. Thus, any extrapolation that derives from cruise reports will necessarily be an underestimate.

Estimates of sample completeness are also derived from an assumption of random sampling, of which these cruise reports are not, and while they serve as a reasonable estimate for relative completeness within this dataset, they are not necessarily of sufficient rigor for comparative analysis across datasets. While family-level richness studies have been shown to be good proxies for species-level biodiversity, they do not account for regions of exceptionally high diversification within lower-level taxonomic groupings. While analysis of sampling completeness can serve as a useful guide for identifying undersampled regions, particularly for managers working off of incomplete data, they should be cautious if attempting to conclude that no additional sampling is need.

Several known hydrothermal vents fields, particularly those associated with mid-plate volcanic hotspots like the Hawai’ian archipelago (Karl et al., 1988), as well as shallow-water vents fields (Tarasov et al., 2005), freshwater vent systems (Crane, Hecker & Golubev, 1991), and submerged volcanoes like Kick’em Jenny in the Caribbean (Carey et al., 2016), are missing from this data set. This is due to both relatively less research conducted in these regions as well as the general unavailability of cruise reports. While these systems represent potential additional targets to further fill knowledge gaps in the deep sea, we do not believe their exclusion substantively impacts the results of our analysis.

This study further highlights the difficulty in accessing biogeographic data from deep-sea hydrothermal vents. Many cruise reports were inaccessible either through proprietary restrictions or archive degradation. While the Census of Marine Life undertook the tremendous task of compiling three decades of biogeographic data through Ocean Biogeographic Information Systems and ChEssBase, that database is now over a decade out of date, and lacks major recent discoveries. With the forthcoming UN Decade of Ocean Science (Intergovernmental Oceanographic Commission, 2018) promoting a renewed focus on global ocean exploration and discovery, a concerted effort to update these databases with contemporary data is warranted.

As new technologies emerge that blur the concept of a research cruise—autonomous drone deployments, cabled observatories, and long-term monitoring platforms—the idea of a research cruise as a discrete sampling unit may no longer be relevant in the next decade of ocean discovery. Instead a continuously updated, shared, and accessible repository of biodiversity data from deep-sea hydrothermal vents is essential for continued monitoring of changes to these ecosystems as they are exposed to increased anthropogenic disturbance.

### Implications for deep-sea mining

The exploration, protection, and potential exploitation of deep-sea hydrothermal vents mirrors a trend common in modern mineral extraction, where prospecting is heavily focused in certain provinces in the Southern Hemisphere, while greater knowledge and understanding has led to more protection in the Northern Hemisphere (Gould, Pellow & Schnaiberg, 2004). Baseline biodiversity data is critical to effective management as it sets the context against which all potential impacts from anthropogenic activities can be assessed. It is particularly concerning that the biogeographic provinces with the highest estimated biodiversity lie in the southern hemisphere, representing tremendous potential for new discovery while simultaneously facing the most imminent threat from deep-sea mining of seafloor massive sulfides.

Due to complex geological, chemical, and physical parameters, no hydrothermal vent system is identical, leading to variation in community composition across vent fields. Communities differ between ocean basins, within ocean basins, and even on much smaller scales (e.g., within a few kilometers: Thaler et al., 2017). As a result, generalizations about hydrothermal vents and their communities are tenuous and the disproportionate representation of northern hemisphere hydrothermal vent ecosystems in the scientific literature could hinder effective management and mitigation policies if used to inform management and mitigation at southern hemisphere hydrothermal vent-derived ore deposits. For example, nearly all studies of community recovery and succession following catastrophic disturbance at a deep-sea hydrothermal vent, which could provide proxies for the impact of and recovery after deep-sea mining, have been conducted at northerly vents on the East Pacific Rise and Juan de Fuca Ridge (Gollner et al., 2015; Marcus, Tunnicliffe & Butterfield, 2009; Mullineaux et al., 2010, 2012; Shank et al., 1998; Tunnicliffe et al., 1997), both of which are mid-ocean ridges. This is problematic as the majority of proposed mining activities are located in the Southwest Pacific back-arc basin, although one recent succession study is available from the Eastern Lau Spreading Center in the Southwest Pacific (Du Preez & Fisher, 2018; Sen et al., 2014). The dramatic difference in biodiversity and abiotic factors such as hydrothermal vent fluid chemistry between these two regions ensure that managers are unable to make direct comparisons between recovery rates in the Eastern and Western Pacific.

This disparity can also have a substantial impact on the effectiveness of regional environmental management plans and set asides (areas protected from mining and secondary impacts that have the potential to act as refugia and larval sources for affected vent communities) to preserve the biodiversity of hydrothermal vent systems (Boschen et al., 2016).Whether or not a set aside will act as an effective buffer against catastrophic disturbance at nearby mining sites depends on many factors, including the resilience of the overall region and the extent to which vent communities are connected (Suzuki et al., 2018). This varies considerably between ocean basins but also within a region. For example, in the Western Pacific, there is extensive regional variability in the ability of vents to recover from disturbance on short time scales. In simulations, Northwest Pacific vent ecosystems tend to have recovery time estimates in the range of 20 years to in excess of a century, while in the Southwest Pacific, recovery times were much shorter, some vents were even predicted to recover within 5 years of mining disturbance (Suzuki et al., 2018).

## Conclusions

Undoubtedly deep-sea mining has the potential for far-reaching impacts on our oceans, both shallow and deep, that could reshape the seafloor for decades, centuries, or longer. Habitat will be removed, sediment plumes will be created, and some biodiversity loss is inevitable (Van Dover et al., 2017). A fundamental problem for predicting the impacts of deep-sea mining on hydrothermal vents is our limited knowledge of these ecosystems in general (Gollner et al., 2017). Hydrothermal vent biodiversity in most regions, especially in the global south, have not been fully characterized. New species are discovered and important ecological insights emerge on every expedition. Recent studies revealed that hydrothermal vent communities in the EEZ of the Kingdom of Tonga are stable over decadal timescales (Du Preez & Fisher, 2018) and that hydrothermal vent ecosystems can act as nursery grounds for non-vent species (Salinas-de-León et al., 2018). There is a lack of basic ecological information, especially for smaller fauna, on population size, behavior, distribution, life history, growth rate, connectivity, and function (Mullineaux et al., 2018). Given the advancement of the nascent deep-sea mining industry, research should accelerate and existing and future data must be made more readily available to the broader deep-sea research community.

## Supplemental Information

10.7717/peerj.7397/supp-1 Supplemental Information 1 Archive of biological cruises to hydrothermal vents. Columns from left to right: Cruise ID, Vessel, Institute, Country, Year, Start Date, End Date, Hemisphere (N/S), Hemisphere (E/W), Ocean/Sea, Putative Biogeographic Province, Geologic Structure, Chief Scientist, Institution, Country, Cruise Report Accessible?, File Name or Hyperlink, Timestamp.

10.7717/peerj.7397/supp-2 Supplemental Information 2 Family incidence data from hydrothermal vent research cruises. Columns 1–3: Phylum, Class, Family. Remaining columns are for each individual cruise report. Each sheet represents a differenet biogeographic province.

10.7717/peerj.7397/supp-3 Supplemental Information 3 Family richness in the Mid-Atlantic Ridge, Northern East-Pacific Rise, and Southwest Pacific sub-divided by phyla (Annelida, Arthropoda, and Mollusca). Parametric interpolation (solid line terminating in black dot) and non-parametric asymptotic extrapolation (dashed line) with 95% confidence intervals (vertical black lines).

10.7717/peerj.7397/supp-4 Supplemental Information 4 Family richness in the Mid-Atlantic Ridge, Northern East-Pacific Rise, and Southwest Pacific for a subset of research cruises ending in 2005 for comparison with ChEssBase. Parametric interpolation (solid line terminating in black dot) and non-parametric asymptotic extrapolation (dashed line) with 95% confidence intervals (vertical black lines).

## Additional Information and Declarations

### Data Availability

The following information was supplied regarding data availability: The raw data is available in the Supplemental Files.
