# Supplementary figures and images for: 262 Voyages Beneath the Sea: a global assessment of macro- and megafaunal biodiversity and research effort at deep-sea hydrothermal vents
Source: PeerJ. 2019 Aug 6;7:e7397. doi: 10.7717/peerj.7397 (PMC6688594; doi:10.7717/peerj.7397)

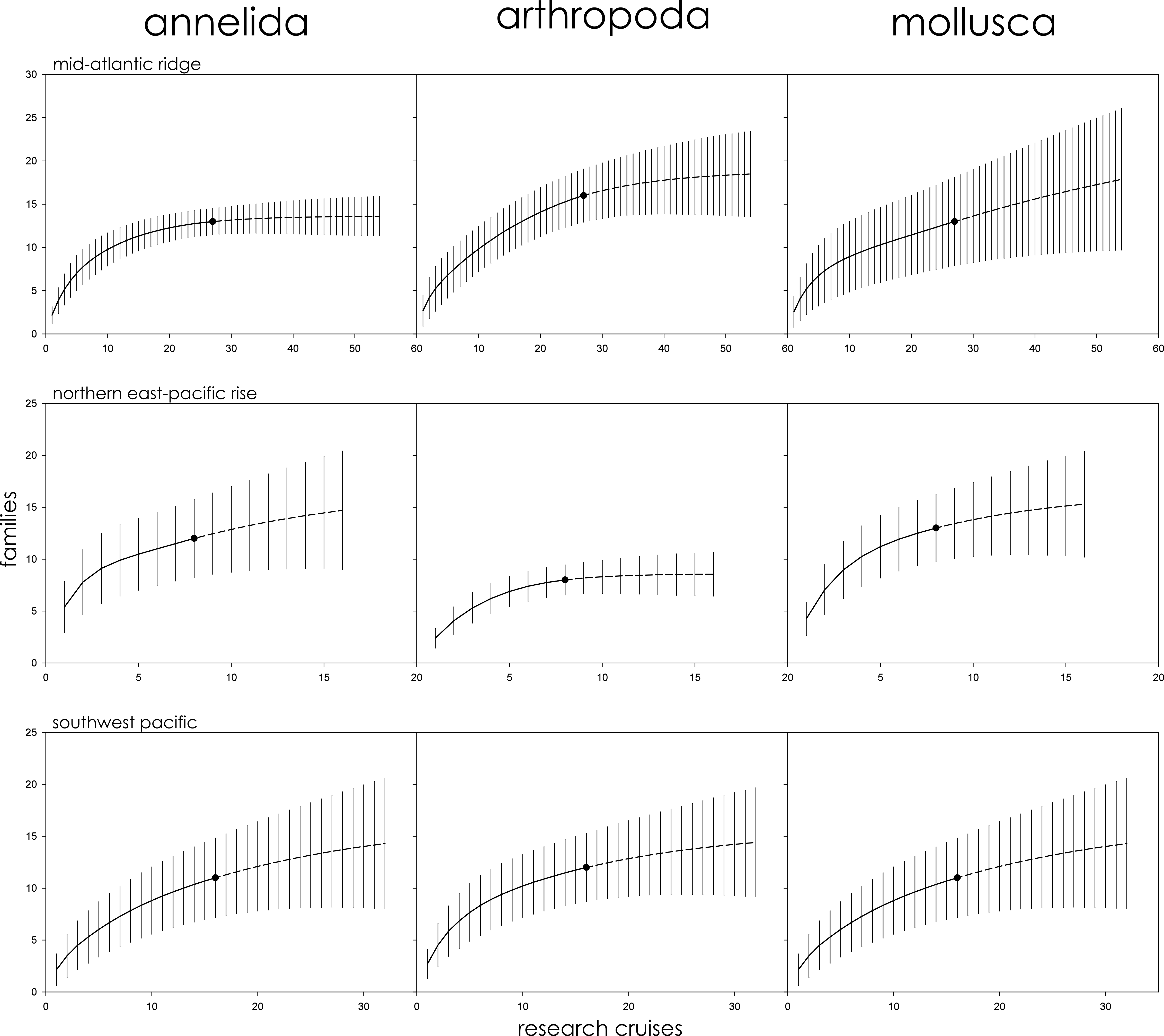

Supplement: Supplemental Information 3 — Parametric interpolation (solid line terminating in black dot) and non-parametric asymptotic extrapolation (dashed line) with 95% confidence intervals (vertical black lines). [file peerj-07-7397-s003.png]

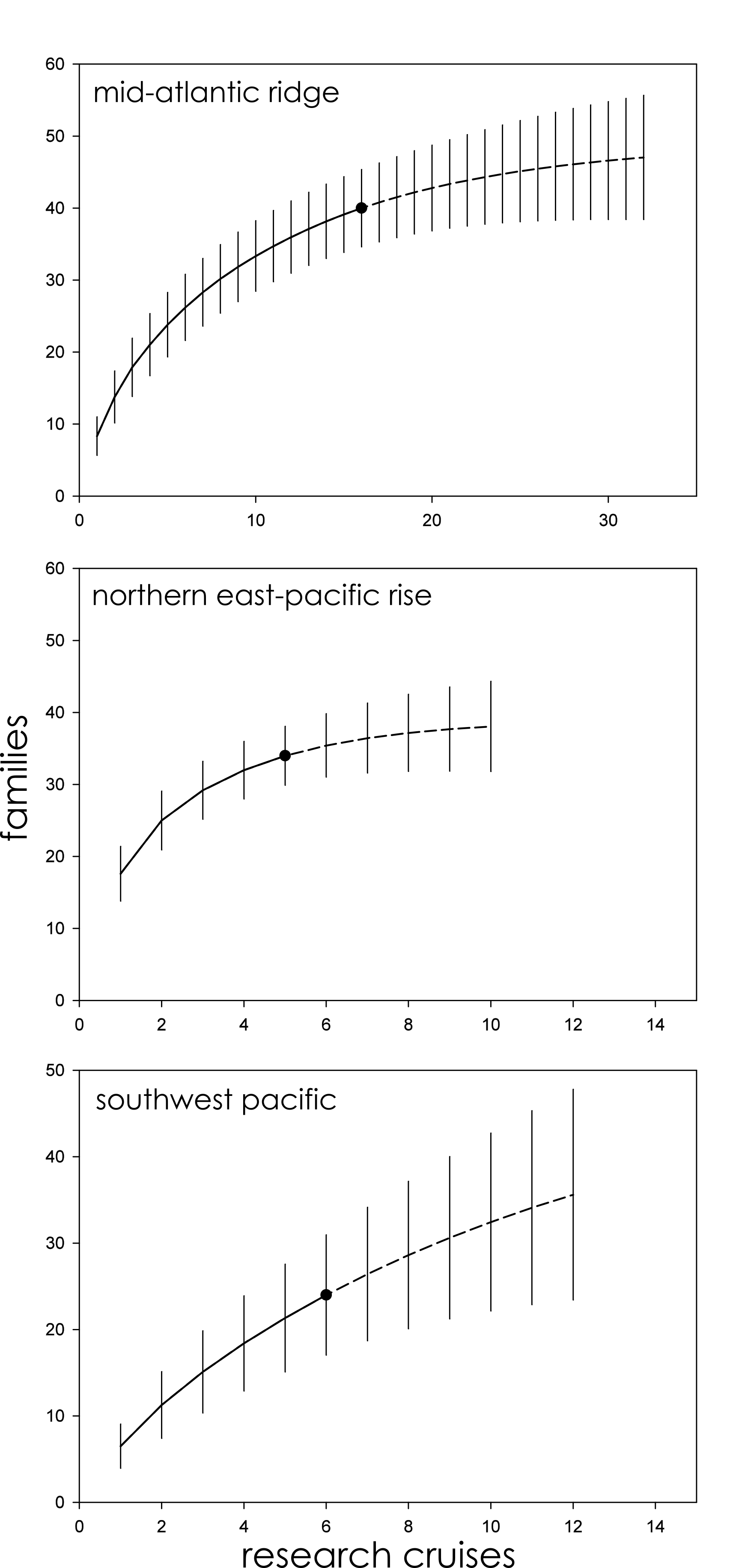

Supplement: Supplemental Information 4 — Parametric interpolation (solid line terminating in black dot) and non-parametric asymptotic extrapolation (dashed line) with 95% confidence intervals (vertical black lines). [file peerj-07-7397-s004.png]
